# Association between Ustekinumab Trough Levels, Serum IL-22, and Oncostatin M Levels and Clinical and Biochemical Outcomes in Patients with Crohn’s Disease

**DOI:** 10.3390/jcm13061539

**Published:** 2024-03-07

**Authors:** Luisa Bertin, Brigida Barberio, Alessandro Gubbiotti, Lorenzo Bertani, Francesco Costa, Linda Ceccarelli, Pierfrancesco Visaggi, Giorgia Bodini, Andrea Pasta, Renato Sablich, Maria Teresa Urbano, Antonio Ferronato, Andrea Buda, Manuela De Bona, Giulio Del Corso, Alessandro Massano, Imerio Angriman, Marco Scarpa, Fabiana Zingone, Edoardo Vincenzo Savarino

**Affiliations:** 1 Gastroenterology Unit, Department of Surgery, Oncology and Gastroenterology, Azienda Ospedale Università Padova, 35128 Padova, Italy; luisa.bertin.1@studenti.unipd.it (L.B.); ale95.massano@gmail.com (A.M.); fabiana.zingone@unipd.it (F.Z.); edoardo.savarino@unipd.it (E.V.S.); 2 Department of General Surgery and Gastroenterology, Tuscany Northwest ASL—Pontedera Hospital, 56025 Pontedera, Italy; 3 IBD Unit, Department of General Surgery, Pisa University Hospital, 56124 Pisa, Italyceccarellilinda@gmail.com (L.C.); pierfrancesco.visaggi@gmail.com (P.V.); 4 Gastrointestinal Unit, Department of Internal Medicine, University of Genoa, 16132 Genoa, Italy; bodini.giorgia@gmail.com (G.B.); andreapasta93@gmail.com (A.P.); 5 Gastroenterology Unit, Santa Maria Degli Angeli Hospital, 33170 Pordenone, Italy; renato.sablich@asfo.sanita.fvg.it (R.S.); mariateresa.urbano@asfo.sanita.fvg.it (M.T.U.); 6 Endoscopy Unit, Department of Medicine, ULSS7 “Pedemontana”, “Alto Vicentino” Hospital, 36014 Santorso, Italy; antonio.ferronato@aulss7.veneto.it; 7 Gastroenterology Unit, Department of Gastrointestinal Oncological Surgery, S. Maria del Prato Hospital, 32032 Feltre, Italy; andrea.buda66@gmail.com (A.B.); manueladebona.md@gmail.com (M.D.B.); 8 Institute of Information Science and Technologies “A. Faedo”, National Research Council of Italy (CNR), 56124 Pisa, Italy; 9 Third Surgical Clinic Section, Department of Surgical, Oncological and Gastroenterological Sciences, Azienda Ospedale Università Padova, 35128 Padova, Italy; imerio.angriman@unipd.it (I.A.); marco.scarpa@aopd.veneto.it (M.S.)

**Keywords:** inflammatory bowel disease, Crohn’s disease, Oncostatin M, IL-22, ustekinumab trough levels, ustekinumab

## Abstract

**Background:** Ustekinumab (UST) has demonstrated effectiveness in treating patients with Crohn’s disease. Monitoring treatment response can improve disease management and reduce healthcare costs. We investigated whether UST trough levels (TLs), serum IL22, and Oncostatin M (OSM) levels could be early indicators of non-response by analysing their correlation with clinical and biochemical outcomes in CD. **Methods:** Patients with CD initiating UST treatment from October 2018 to September 2020 were enrolled at six Italian centres for inflammatory bowel disease (IBD). Clinical and biochemical data were collected at four time points: baseline, second subcutaneous (SC) dose, fourth SC dose, and 52 weeks. TLs were measured during maintenance, at the second SC dose, and at the fourth SC dose. IL-22 and OSM serum levels were assessed at baseline and the second SC dose. We analysed whether TLs, IL22 levels, and OSM serum levels were associated with clinical response, clinical remission, biochemical remission, and endoscopic remission using the appropriate statistical tests. **Results:** Out of eighty-four initially enrolled patients, five were lost to follow-up, and eleven discontinued the drug before 52 weeks. At the 52-week time point, 47% achieved biochemical remission based on faecal calprotectin levels, and 61.8% achieved clinical remission. TLs at the second SC dose significantly correlated with biochemical remission at the same time point (*p* = 0.011). However, TLs did not correlate with clinical remission. Baseline OSM levels did not correlate with biochemical or clinical remission or response. IL22 levels notably decreased during UST therapy (*p* = 0.000), but its values did not correlate with biochemical or clinical remission. **Conclusions:** UST is an effective therapy for patients with CD. TLs measured at the second SC dose significantly correlated with biochemical remission, emphasising their potential role in treatment monitoring. Levels of OSM and IL-22, despite a significant decrease in the latter during therapy, did not exhibit correlations with clinical or biochemical outcomes in our study. Further studies are needed to confirm these findings.

## 1. Introduction

Crohn’s disease (CD) is a chronic inflammatory disorder of the gastrointestinal tract that affects millions of individuals worldwide [1]. CD can affect any part of the gastrointestinal tract, with the ileocaecum being the most commonly affected site [2]. While some patients experience a mild disease course, most face a relapsing and remitting pattern with progressive intestinal damage that may require surgery [3]. 

The management of CD is multifaceted and depends on disease location, severity, and response to previous treatments [4]. The primary goal of therapy is to induce and maintain remission as well as improve patients’ quality of life [4]. Obtaining enduring, profound remission is crucial to avert permanent gastrointestinal harm and disability [5,6]. Therapies for CD revolve around four primary mechanisms of action: agents that target anti-tumour necrosis factor (anti-TNF), such as infliximab and adalimumab; therapies against anti-interleukin, including ustekinumab and risankizumab; treatments involving anti-integrin, such as vedolizumab; and immunomodulators, commonly prescribed alongside anti-TNF agents [7,8,9].

Ustekinumab (UST, marketed as Stelara; Janssen Biotech, Inc, Horsham, PA, USA) represents a fully human IgG1 monoclonal antibody designed to target the shared p40 subunit of interleukin 12 (IL12) and 23 (IL23) and effectively inhibit the T-helper 1 (Th1) and T-helper 17 (Th17) pathways, which play crucial roles in CD [8,10,11,12,13,14]. UST has shown favourable results in real-life studies [15,16,17,18] and a good safety profile [19,20,21,22,23,24,25]. 

In the current scenario, where multiple new compounds with different mechanisms of action have recently been licensed for use in inflammatory bowel disease (IBD) or are in late-stage trials, it is not feasible to ascertain the treatment that is most probable to yield effectiveness while minimising the potential for toxicity in an individual patient [26,27]. Currently, endeavours are underway to integrate precision medicine into IBD management, taking inspiration from the approaches employed by oncologists. To achieve remission in a broader cohort, further efforts are warranted, particularly in the pursuit of biomarkers capable of predicting the efficacy of a particular drug [28,29,30]. Until now, the treatment approach has often relied on clinical tools with limited predictive accuracy [31], and potential biomarkers are under investigation primarily in cases of anti-TNF treatment failure [28,32]. 

Moreover, customising dosages to optimise treatment responses in IBD presents an encouraging avenue through the application of therapeutic drug monitoring (TDM) [33]. Reactive TDM involves assessing drug levels and antibodies in response issues in primary non-response or secondary loss of response to therapy. At the same time, proactive TDM regularly monitors drug concentrations and adjusts doses to maintain optimal levels. The utility of TDM in clinical application is well established, particularly concerning anti-TNF medications [34]. Nevertheless, there is a lack of data to guide clinicians in establishing target drug-level thresholds when treating patients with CD undergoing UST treatment. Currently, the optimal range for maintaining UST levels has not been definitively established, necessitating further research to elucidate the impact of TDM on disease monitoring and treatment strategies. Extensive data from retrospective and prospective studies and post hoc analyses of randomised controlled trials (RCTs) demonstrate that elevated drug concentrations in IBD are linked to enhanced therapeutic outcomes [35].

So, our research sought to explore the significance of potential biomarkers like IL-22 and Oncostatin M (OSM) in individuals with CD who underwent UST treatment, assessing how they relate to treatment outcomes and safety. Additionally, we examined Ustekinumab trough levels (TLs) to determine their potential in guiding clinicians’ management of CD patients.

## 2. Materials and Methods

### 2.1. Population and Study Design

This multicentre prospective cohort study was coordinated by the IBD Unit of Padua University, involving 5 other Italian IBD centres (Feltre, Genova, Pisa, Pordenone, Santorso).

From October 2018 to September 2020, adult patients with moderate-to-severe CD who started therapy with UST were prospectively enrolled. We excluded patients who had undergone surgical intervention within the last 12 months before study enrolment. 

As standard clinical practice, patients received a single intravenous (IV) induction of UST, with dosing depending on body weight: 260 mg if the patient weighed 55 kg or less, 390 mg if the patient weighed between 55 and 85 kg, and 520 mg if the patient weighed more than 85 kg. Afterwards, the patients received 90 mg of subcutaneous (SC) UST after eight weeks. Maintenance was carried out with subcutaneous UST administration every eight weeks (Q8W) or every 12 weeks (Q12W).

After obtaining informed consent, venous blood samples were collected in covered test tubes (Vacutainer SST II Advance, Roborough, Plymouth, UK). Tubes containing whole blood were left undisturbed at room temperature for 15–30 min and then centrifugated at 3000× *g* for 15 min. Each tube’s resulting supernatant (serum) was stored at −20 °C until analysis. UST TLs were evaluated at the second SC infusion and the fourth SC infusion, while IL22 levels and OSM serum levels were dosed at baseline and the second SC dose. Patients were prospectively evaluated, and the following parameters were obtained during each follow-up evaluation: gender, age, disease duration, disease location, disease behaviour, smoking status, previous treatments, indication for UST treatment, Harvey–Bradshaw index (HBI), Simple Endoscopic Score for Crohn’s Disease (SES-CD), concomitant prescription of immunosuppressive therapies or corticosteroids, optimisation of UST treatment, hospitalisations related to CD, and surgical interventions. All adverse events (AEs), not only those that led to discontinuation of therapy, were recorded. Severe AEs were defined as those requiring hospitalisation or therapy discontinuation. Blood levels of C-reactive protein (CRP) and faecal calprotectin (FC) values were also collected at baseline, at the second and fourth SC injections, and at 52 weeks. An endoscopic assessment was performed at week 52. 

**Figure 1:**
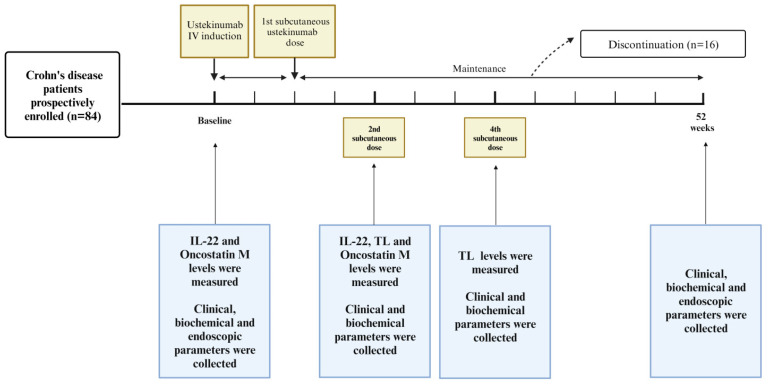
Study design. Created with Biorender.com. Last accessed on 23 January 2024. From October 2018 to September 2020, adult patients with moderate-to-severe CD initiating therapy with UST were prospectively enrolled at 6 Italian centres for the treatment of IBD. As per standard clinical practice, patients received a single intravenous induction of UST, and subsequently, patients received 90 mg of subcutaneous UST after eight weeks, followed by maintenance therapy administered every eight weeks or twelve weeks. UST TLs were evaluated at the second and fourth SC infusions, while serum IL22 and OSM levels were assessed at baseline and the second SC dose. Clinical and biochemical parameters, including C-reactive protein and faecal calprotectin levels, were collected at baseline, second and fourth SC injections, and at 52 weeks. Additionally, an endoscopic assessment was conducted at week 52.

The study design is illustrated in Figure 1.

### 2.2. Definition and Outcomes

According to the medical literature, we defined clinical remission as HBI ≤ 5, steroid-free clinical remission as HBI ≤ 5 without steroids use, and clinical response as more than 3 points’ reduction in baseline HBI score with a concomitant decrease in steroid dosage until its discontinuation within eight weeks [36,37,38]. Biochemical remission was defined as CRP levels below 5 mg/L throughout the follow-up period and/or FC ≤ 250 μg/g [39]. Endoscopic remission was defined as an SES-CD score < 3 [40]. Treatment failure was defined as discontinuation of biological therapy due to AEs, lack of clinical response, and need for hospitalisation/surgery. Finally, we assessed the relationship between clinical and biochemical responses to UST treatment and serum UST TLs, IL-22, and OSM levels.

### 2.3. Measurement of Faecal Calprotectin Levels

FC was measured using a commercial quantitative ELISA (Calprest, Dynex Elisa Eurospital, Trieste, Italy). Faecal samples were diluted 1:250 in the kit-recommended diluent buffer. FC levels are expressed as μg/gram of wet faeces.

### 2.4. Measurement of CRP

Serum CRP concentrations were measured using a commercial enzyme-linked immunosorbent assay kit (apDia, Belgium, cat. N° 740011) able to recognise circulating CRP. Serum samples were diluted 1:1000, according to the manufacturer’s instruction, and incubated into a microplate precoated with a monoclonal antibody specific for human CRP. As suggested, sample concentrations were retrieved by generating a linear curve fit using GraphPad Prism v.9 (GraphPad Software, San Diego, CA, USA). The minimum detectable concentration was ∼0.02 µg/mL. Intra-assay and inter-assay coefficients of variation ranged from 4.1% to 6.9% and 5.8% to 6.3%, respectively. A value of CRP ≥ 5 mg/L was considered abnormal.

### 2.5. Measurement of Serum UST Trough Levels

Trough levels of UST were quantified using a specific biotinylated antibody directed against the idiotype of UST in an enzyme-linked immunosorbent assay (ELISA), according to the manufacturer’s protocol (ImmunoGuide, AybayTech Biotechnology, Ankara, Turkey). This assay can measure UST TLs from 0.04 to 1.00 µg/mL with a 2 ng/mL detection limit. Blood was collected before the next dose (trough concentration). 

### 2.6. Cytokine Analysis

For the quantification of IL-22 and OSM in human serum samples, the human IL-22 ELISA Kit (Raybiotech Inc., Peachtree Corners, GA, USA) and Human Oncostatin M Elisa kit (EHOSM) (Thermo-Fisher-Scientific, Waltham, MA, USA) were used following the manufacturer’s guidelines. Results are expressed as picogram per millilitre.

All blood samples and results were collected and analysed blindly until the end of the study. Biologists performing the analysis of UST TLs were blinded to the clinical, endoscopic, and biochemical data of UST-treated patients.

### 2.7. Statistical Analysis

Data were analysed using STATA 11.1 software (Stata Corp., College Station, TX, USA) and IBM SPSS Statistics software version 26 (IBM Corp., Armonk, NY, USA). Continuous variables are reported as medians with interquartile ranges from 25% to 75%, and categorical variables are reported as frequencies and percentages. The differences between the independent groups were evaluated using Student’s *t*-test or the Mann–Whitney exact test, as appropriate. To determine if there is a statistically significant difference in proportion between paired data, we used McNemar’s test. At the same time, comparison between ordinal or continuous values over the study period was performed using the Wilcoxon signed-rank test. Statistical significance was set for values of *p* ≤ 0.05.

## 3. Results

### 3.1. Baseline Characteristics

Overall, eighty-four patients with CD treated with UST were enrolled, of whom five were lost to follow-up, and eleven discontinued the drug before 52 weeks. Their baseline characteristics are presented in Table 1. Most of the patients were men (61.9%). Among the included patients, only three patients (3.5%) were anti-TNF-naïve due to contraindications to its use. The median baseline HBI score was 5. At study initiation, 22.6% of patients were receiving concomitant corticosteroids. Median FC values at baseline were 1000 µg/g, IQR: 452–1900. As for endoscopic parameters, the median SES-CD score was 7 (IQR: 3–13). Forty-six patients (54.8%) had had prior intestinal resection.

### 3.2. Clinical, Biochemical, and Endoscopic Outcomes

#### 3.2.1. Clinical Outcomes

By the second SC dose, 57 patients (67.8%) were in clinical remission (*p* = 0.007), and 50 patients (59.5%) were in steroid-free clinical remission (*p* = 0.017). Upon reaching the fourth infusion, a total of 52 patients (61.9%) were in clinical remission (*p* = 0.002), and among these, 46 patients (54.7%) were also in steroid-free clinical remission (*p* = 0.003). By the 52nd week, 42 patients (61.7%) had achieved clinical remission (*p* = 0.009). Among these, 41 patients (60.2%) were in steroid-free clinical remission (*p* = 0.001). 

#### 3.2.2. Biochemical Outcomes

By the second and fourth SC injections, 13 (15.47%) and 27 patients (32.1%) had achieved biochemical remission based on FC levels. Additionally, at 52 weeks, 32 patients (47.0%) demonstrated negative values for FC. From baseline to the second SC dose, we found a statistically significant reduction in median FC values (1000 µg/g, IQR: 452-1900 µg/g vs. 330 µg/g, IQR: 115-1260, *p* < 0.0001). Subsequently, the median FC value was significantly lower compared to baseline at the fourth SC dose (461 µg/g, IQR: 119-1428, *p* = 0.035) and 52 weeks (244 µg/g, IQR: 82–798, *p* = 0.036). Initially, 46 patients (54.7%) had negative CRP values; the number increased to 55 patients (65.4%) in biochemical remission according to PCR levels at the fourth SC dose (*p* = 0.041). This trend persisted through week 52, with 47 participants (69.1%) maintaining biochemical remission according to PCR levels (*p* = 0.017). 

#### 3.2.3. Endoscopic Outcomes

At week 52, 52 patients (68.0%) were in endoscopic remission. As for the baseline, the median SES-CD score significantly improved, with a value of 3 (IQR:1–5; *p* < 0.001) at week 52.

**Figure 2:**
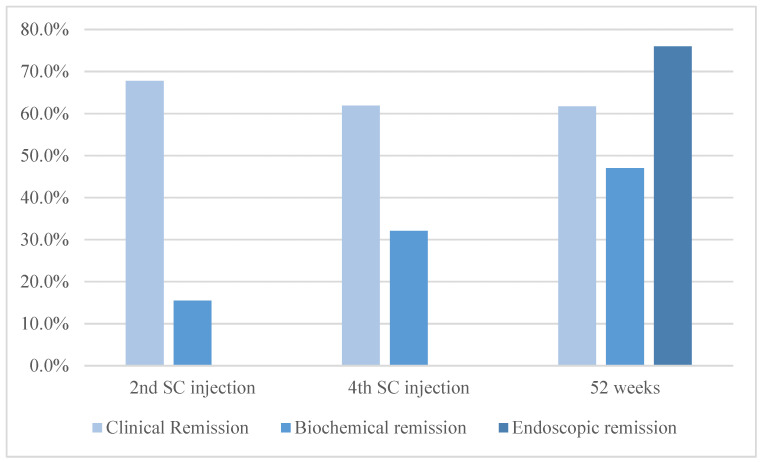
Clinical, biochemical, and endoscopic remission in patients with CD undergoing ustekinumab therapy. The light shade signifies clinical remission, while medium blue indicates biochemical remission, and dark blue denotes endoscopic remission, all as defined within the paper. The figure displays the outcomes of ustekinumab therapy in patients with CD. By the second SC dose, 67.8% had achieved clinical remission, slightly decreasing to 61.9% by the fourth dose and 61.7% by week 52. Based on FC levels, biochemical remission rates were 15.47% in the second and 32.1% in the fourth dose. By week 52, 47.0% demonstrated negative FC values. Endoscopic remission had been achieved in 68.0% of patients by week 52, with a significant improvement in SES-CD score from baseline.

All the outcomes of the population under study are represented in Figure 2.

### 3.3. Optimisation of Ustekinumab Dosing

Most patients had UST optimised to Q8W at the second SC dose, and the percentage gradually increased from 73% to 81%. Therefore, 27% of patients continued having UST administered Q12W at the second SC dose (*n* = 22) and 25% (*n* = 21) at the fourth SC dose; by week 52, the number of patients still maintaining this dosing schedule had reduced to 19% (*n* = 13; *p* = 0.092). There were no statistically significant differences observed in the achievement of either clinical remission (*p* = 0.261) or steroid-free clinical remission (*p* = 0.338) between the two dosing intervals (Q8W and Q12W). Furthermore, there was no discrepancy in attaining biochemical remission (*p* = 0.182). There was no significant difference in the occurrence of adverse events between patients who followed the two distinct dosing schedules at various time points (*p* = 0.279; *p* = 0.272; *p* = 0.218).

### 3.4. Safety Profile and Drug Discontinuation

In total, fourteen AEs were reported, ten mild and four severe. The types of AEs are described in the Supplementary Files, Table S1. Among the eighty-four enrolled patients, five were lost to follow-up and eleven discontinued the medication during the 52-week follow-up period. Among the latter, nine discontinued the drug due to lack of effectiveness, one due to colic perforation and abscess formation, and one stopped the treatment due to an allergic cutaneous reaction.

### 3.5. Ustekinumab Trough Levels

**Figure 3:**
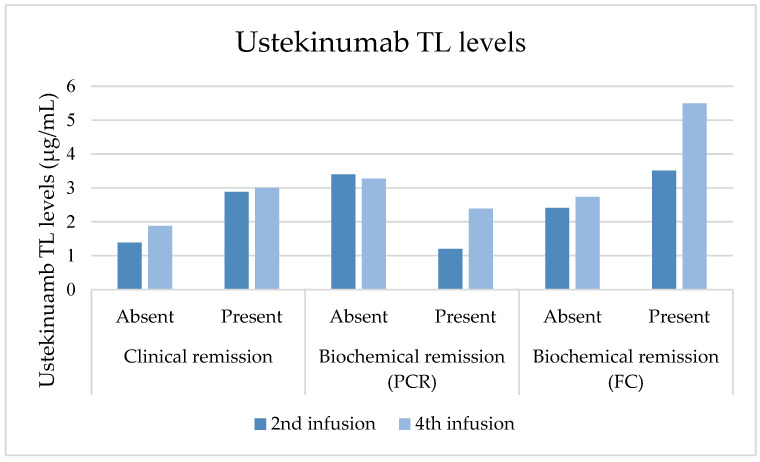
Comparison of ustekinumab TLs at second and fourth SC dose with clinical and biochemical outcomes at those time points. This figure illustrates the comparison of ustekinumab TLs at the second and fourth SC doses with clinical and biochemical outcomes at those time points. At the second and fourth SC dose, patients achieving steroid-free clinical remission had higher TLs (3.11 μg/mL ± 2.03 and 4.08 μg/mL ± 3.46) compared to those who did not (2.28 μg/mL ± 1.74, *p* = 0.216 and 2.71 μg/mL ± 2.51, *p* = 0.367). At the second SC dose, those achieving biochemical remission based on FC values had higher average TLs (3.51 μg/mL ± 1.65 and 5.49 μg/mL ± 4.22) compared to those who did not (2.41 μg/mL ± 2.05, *p* = 0.011 and 2.73 μg/mL ± 2.16, *p* = 0.165). On the contrary, UST TLs were lower in patients with a negative PCR at the second and fourth SC doses.

The median TLs of UST at the second SC dose were 2.89 μg/mL (IQR 1.12–3.99), decreasing to 2.45 μg/mL (IQR 1.33–6.70) at the fourth SC dose, although not significantly (*p* = 0.263). Those achieving steroid-free clinical remission at the second SC dose had higher TLs (3.11 μg/mL ± 2.03) compared to those who did not (2.28 μg/mL ± 1.74, *p* = 0.216). Similarly, those achieving biochemical remission based on FC values at the second SC dose had higher average TLs (3.51 μg/mL ± 1.65) compared to those who did not (2.41 μg/mL ± 2.05, *p* = 0.011). At the fourth SC dose, patients in steroid-free clinical remission had higher mean TLs (4.08 μg/mL ± 3.46) than those who were not (2.71 μg/mL ± 2.51, *p* = 0.367). Similarly, those in biochemical remission based on FC value had higher mean TLs (5.49 μg/mL ± 4.22) compared to those who were not (2.73 μg/mL ± 2.16, *p* = 0.165). UST TLs compared to outcomes are represented in Figure 3. Clinical and biochemical outcomes at the fourth SC dose are depicted in Supplementary files, Table S2.

TL concentrations at the second and fourth SC doses were similar between patients using immunosuppressants or steroids and non-users. No notable differences in TLs were observed between patients experiencing AEs and those who did not by the fourth SC dose. Logistic regression analysis showed no correlation between UST TLs and AEs at the second and fourth SC doses. TLs at the fourth SC dose were not influenced by interval dosing, and there were no significant differences in TLs among patients who underwent resection surgery during the study period. 

### 3.6. Oncostatin M Plasma Values

**Figure 4:**
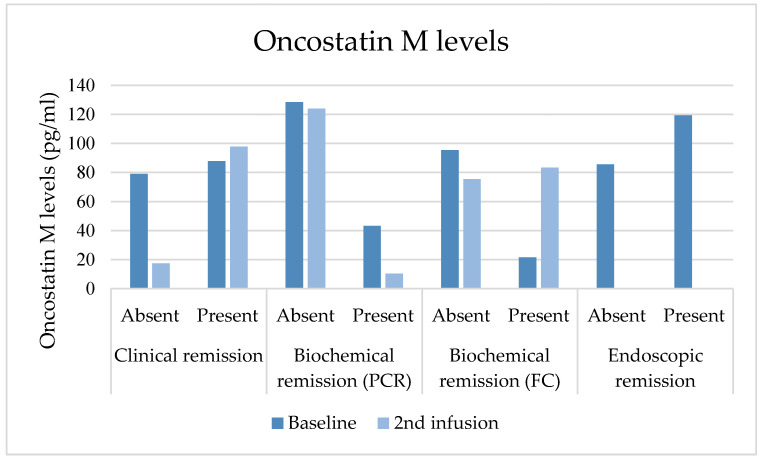
Comparison of Oncostatin M at baseline and second SC dose with clinical and biochemical outcomes and endoscopic evaluations at baseline. This figure presents a comparison of OSM levels at baseline and the second SC dose with clinical and biochemical outcomes at baseline, as well as endoscopic evaluations at baseline. At the second SC dose, median OSM was higher in patients achieving steroid-free clinical remission and those in biochemical remission based on FC. However, the differences were not statistically significant (*p* = 0.1597; *p* = 0.169). No other significant correlations were found when compared to outcomes.

Baseline median OSM was 112.09 pg/mL (IQR = 46.0–312.9) and decreased to 67.5 pg/mL (IQR = 33.6–365.1; *p* = 0.889) at the second dose. At the second SC dose, median OSM was higher in patients in steroid-free clinical remission and patients with biochemical remission based on FC (*p* = 0.1597; *p* = 0.169). There was no correlation between baseline OSM levels and those at the second SC dose and clinical response or steroid-free clinical remission. OSM levels compared to outcomes are represented in Figure 4.

### 3.7. IL-22 Plasma Values

**Figure 5:**
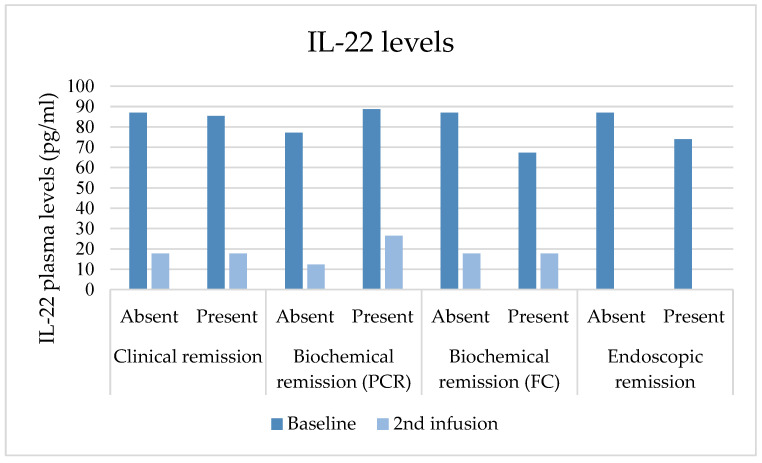
Comparison of IL-22 at baseline and second SC dose with clinical and biochemical outcomes and endoscopic evaluations at baseline. This figure depicts the comparison of IL-22 levels at baseline and the second SC dose with clinical and biochemical outcomes at baseline and endoscopic assessment at baseline. IL-22 levels at the second SC dose were lower in patients with biochemical remission based on FC, although without a statistically significant difference (*p* = 0.528). No other significant correlations were found when compared to outcomes.

Median IL-22 levels significantly decreased from baseline to the second SC dose (86.19 pg/mL, IQR 65.71–94.79 vs. 17.72 pg/mL, IQR 8.50–26.45; *p* = 0.000). IL-22 levels at the second SC dose were lower in patients with clinical response and biochemical remission based on FC, although without a statistically significant difference (*p* = 0.524; *p* = 0.528). IL-22 plasma values compared to outcomes are represented in Figure 5.

## 4. Discussion

For patients with CD, a personalised approach is critical and successful patient-specific treatment is of paramount importance [41]. Our study examined the levels of IL-22, OSM, and TLs in the serum of patients with CD undergoing ustekinumab therapy, evaluating their role as potential prognostic biomarkers. The pursuit of predictive biomarkers has been fuelled by the expanding range of available treatments and their varying degrees of effectiveness. However, most studies on biomarkers have primarily focused on non-response to anti-TNF therapies [42].

At the second SC dose, 67.8% of patients were in clinical remission (*p* = 0.007), and 60.2% were in steroid-free clinical remission (*p* = 0.017). At 52 weeks, 61.7% of patients were in clinical remission (*p* = 0.009) and 60.2% were in steroid-free clinical remission (*p* = 0.001). UST TLs at the second SC dose significantly correlated with biochemical remission at the same time point (*p* = 0.01) but not with steroid-free clinical remission (*p* = 0.216). At the second SC dose, mean OSM was higher in patients in steroid-free clinical remission and patients in biochemical remission, although not significantly (*p* = 0.1597; *p* = 0.169). Moreover, we found median IL-22 levels to be 86.19 pg/mL (IQR 65.71–94.79) during the initial assessment but significantly decreased to 17.72 pg/mL (IQR 8.50–26.45; *p* < 0.001) after the second SC dose. However, IL-22 plasma levels were not significantly different in those achieving clinical and biochemical remission at that time point.

A recent systematic review and meta-analysis, including 63 real-world studies involving 8529 patients with CD treated with UST, reported varying rates of clinical remission: 37% (28–46%) in the short term, 42% (36–49%) in the medium term, and 45% (37–53%) in the long term [43]. The variations observed in these results may be partially ascribed to the different criteria used to define clinical remission, the clinical assessment’s timing, and UST’s varied dosing schedules. Regarding the first point, the meta-analysis conducted by Rubín de Célix et al. included studies that employed diverse assessment tools, such as Physician Global Assessment (PGA). In contrast, our study exclusively utilised HBI for defining clinical remission. As per the timing of the assessment, Rubín de Célix et al. defined short-term as occurring between 8 and 14 weeks, and in our study, short-term evaluation took place at the second subcutaneous dose. In the previously mentioned meta-analysis, the endoscopic remission rate was 33% over the long term (48 to 52 weeks), which is lower than our findings [43,44]. This disparity could be attributed to our elevated proportion of patients with a history of intestinal resection, where the SES-CD score might underestimate the endoscopic data and the drop-out rate. To our knowledge, the safety of UST in patients with CD in observational studies was recently summarised by Honap et al., where 498 AEs were reported in 2977 patients (17%), resulting in a pooled estimate of the incidence rate of 14% [45]. This aligns with our study, where 15.4% of patients developed AEs, with severe AEs in 4.7% of patients. The proportion of serious AEs in observational real-world studies is low, and only a minority (6.8%) lead to the discontinuation of UST treatment [43]. 

IL-22 is a member of the IL-10 cytokine family, primarily from several immune cell types, with Th1 and Th22 cells being its main contributors [46]. IL-22 is a significant communication mode connecting the immune system with specialised tissue cells, such as the skin, gut, lungs, and liver [46]. The role of IL-22 in IBD is challenging to elucidate. While IL-22 is essential in promoting mucus production and stimulating the proliferation of epithelial cells, its Janus-faced nature has been recognised in inflammatory and autoimmune disorders. Certain studies have indicated that it can stimulate inflammation and tumourigenesis in specific contexts [47,48,49] or that it may contribute to the development of IBD [50,51,52,53]. These conflicting data, however, point towards a protective role of IL-22 in IBD where it could promote epithelial repair. Both recombinant IL-22 and IL-22 agonists are being studied as a therapy in IBD [54,55]. An antibody that blocks IL-23, like UST, should hinder the development and growth of pathogenic Th17 cells dependent on IL-23, resulting in a decrease in cytokines linked to this cell type, including IL-22 [56]. Higher doses of IL-22 plasma levels have been associated with higher disease activity in IBD patients defined with clinical scores in a recent study by the SPARC registry [57]. In clinical trials evaluating the efficacy of risankizumab and brazikumab, two monoclonal antibodies targeting the IL-23 p19 subunit in individuals with moderately-to-severely active CD, IL-22 plasma concentrations were assessed. A pooled analysis of 52 patients with CD who received risankizumab in ADVANCE or MOTIVATE and FORTIFY showed that induction baseline serum IL-22 concentrations were not predictive of week 52 stool frequency, abdominal pain score, clinical remission, clinical response, endoscopic response, or endoscopic remission. In contrast, during the phase 2a INTREPID study, serum IL-22 emerged as a potential biomarker for brazikumab’s efficacy. Following brazikumab induction therapy for CD, IL-22 levels decreased, and patients with IL-22 levels exceeding the median threshold of 15.6 pg/mL were notably more likely to achieve clinical response and remission than the placebo group. The substantial reduction in IL-22 levels observed in our study during UST therapy aligns with results reported in previous clinical trials involving risankizumab and brazikumab. Nevertheless, neither the baseline levels of IL-22 nor those at the second SC dose exhibited a precise predictive capacity for achieving clinical or endoscopic remission. These findings diverge from those reported in a recent brazikumab trial but concur with the results of the risankizumab trial. It is essential to note that our study was prospective, featured a relatively smaller sample size, lacked a placebo group for comparative analysis, and encompassed both Q12W and Q8W UST therapy regimens. As such, these findings necessitate validation through larger cohorts, considering potential patient selection bias. 

OSM is a glycoprotein that belongs to the interleukin-6 family of cytokines and functions mainly in cell growth [58]. OSM can promote tissue repair and support diverse homeostatic processes, but its overproduction is thought to promote a variety of pathologies, including skin and lung inflammation, atherosclerosis, and several forms of cancer [59,60]. The involvement of the OSM-OSMR axis in the pathogenesis of IBD was initially identified by detecting a disease susceptibility single-nucleotide polymorphism (SNP) located within the OSMR locus on chromosome 5 [61]. In the context of serum OSM levels, the available evidence lacks uniformity. Higher serum OSM levels were found in patients who subsequently lost response to infliximab therapy [62,63,64]. However, in this case, data were incoherent, and the cited studies’ sample size was limited [65]. An initial proteomic examination conducted within the extensive PANTS cohort has confirmed that baseline serum levels of OSM remain uncorrelated with the response to anti-TNF therapy in individuals diagnosed with CD [66]. In one study, serum OSM levels before treatment could not predict response to vedolizumab in IBD [65]. Likewise, our investigation reveals a disparity between OSM values and remission status, as defined by clinical, endoscopic, and biochemical criteria. We did not observe any correlation between OSM plasma levels and the reduced efficacy of UST. Further studies on this subject are required, with a larger cohort to confirm and validate these results. 

Various studies have detailed the connection between UST TLs and clinical response in CD. The pharmacokinetic characteristics of UST have initially been delineated in individuals with psoriasis. In the case of patients with CD, the variability in UST clearance is influenced by factors such as body weight, serum albumin concentration, CRP levels, the history of TNF antagonist treatment failure, gender, racial background (Asian versus non-Asian), and the presence or absence of antibodies to UST [67]. Analysis of drug concentrations derived from pivotal induction UNITI clinical trials revealed that a steady state had been achieved by the second SC maintenance dose [68]. Intriguingly, patients with elevated drug concentrations exhibited higher rates of clinical remission, endoscopic improvement, and biochemical remission [68]. The volume of publications about TDM of UST in patients with IBD is rising. Real-world studies have indicated a correlation between UST levels and clinical outcome measures [69,70,71,72,73,74,75], endoscopic outcomes [70,76,77,78,79], or biochemical ones [72,73,74,76,80,81,82]. Although these findings were not confirmed by evidence on clinical outcomes [80,81,82,83] or endoscopic outcomes [80,81], most real-world studies affirm the significance of trough concentrations in CD. It is worth mentioning that subsequent observational studies have individuated higher optimal TL concentrations than those identified in RCTs. Disparities emerge concerning assay methodologies (including ELISA and homogeneous mobility shift assay (HMSA)), dosing intervals, timing of TL dosing at endoscopic, biochemical, and clinical assessment, and definition of remission or response. These discrepancies manifest as contradictions in delineating the ideal therapeutic range across these studies. In our study, median UST TLs were 2.89 μg/mL (IQR 1.12–3.99) at the second SC dose and 2.45 μg/mL (IQR 1.33–6.70) at the fourth SC dose. The TLs in our cohort were higher than the post hoc IM-UNITI analyses. In IM-UNITI, the median concentrations measured just before administration remained consistent from week 8 to week 44 for both the Q8W group (ranging from 2.0 mg/mL to 2.2 mg/mL at IM-UNITI weeks 8, 16, 24, 32, and 40) and the Q12W group (ranging from 0.6 mg/mL to 0.8 mg/mL at IM-UNITI weeks 12, 24, and 36). Average UST concentrations in the Q8W group were approximately three times higher than those in the Q12W group both in the UNITI and IM-UNITI trials [44,68]. In our cohort, the mean levels were not significantly different between the Q12W and the Q8W groups, both at the second SC dose and at the fourth SC dose. In IM-UNITI, there was a tendency towards achieving higher clinical remission rates in the UST Q8W group [44]. Conversely, clinical remission was not associated with dosing interval in our study. However, most of our patients had UST administered Q8W and patients at the two dosing intervals were not matched. It could be inferred that patients with increased disease activity or more significant risk factors were optimised earlier; thus, this outcome needs to be appropriately contextualised. Notably, our study reaffirmed the autonomy of UST TLs from immunomodulators. Unlike anti-TNF-α medications, it appears that the simultaneous utilisation of immunomodulators does not substantially influence UST concentrations.

One of the strengths of our study is that we enrolled a homogenous study cohort consisting of individuals with moderate-to-severe CD exhibiting similar baseline characteristics, including disease duration, medical history, and prior treatments. Moreover, this patient population aligns with those found in other real-world cohorts [12,13,84]. Another noteworthy strength lies in the systematic assessment of UST levels, IL-22, and OSM using ELISA tests. Nonetheless, several limitations must be recognised. These include the relatively small sample size and the exclusive inclusion of Italian patients. Consequently, our study population may not be fully representative of a more diverse global population. The utilisation of the HBI as the sole measure of clinical remission is a limitation, as its usefulness and validity have faced criticism. Additionally, it is essential to emphasise that while our study identifies associations, it cannot establish causation between cytokine analysis and treatment outcomes. A potential limitation of our study is the higher percentage of patients with a history of intestinal resection, which may lead to the underestimation of endoscopic data by the SES-CD score.

## 5. Conclusions

In conclusion, our findings suggest a connection between UST TLs at the second dose and the achievement of biochemical remission in individuals with CD. To ascertain whether treatment outcomes can be enhanced through dose escalation guided by TLs, there is a requirement for prospective randomised trials. Although serum OSM levels at baseline were lower in patients in biochemical remission, these concentrations were not coherently associated with clinical outcomes. IL22 levels significantly lowered during UST therapy but did not correlate with clinical and biochemical remission.

## Figures and Tables

**Table 1:** Baseline characteristics of patients with Crohn’s disease initiating ustekinumab.

		*n* = 84
Age at diagnosis (years)	Median (IQR)	27 (19–40)
Age at inclusion (years)	Mean (SD)	43 (14)
Female	*n* (%)	32 (38.1%)
Disease duration (years)	Median (IQR)	12 (7–21)
Follow-up (months)	Median (IQR)	15 (13–16)
Active smoking	*n* (%)	10 (11.9%)
Disease activity		
SES-CD score	Median (IQR)	7 (3.00–13.00)
HBI score	Median (IQR)	5 (2–9)
CRP above the upper normal limit	*n* (%)	38 (45.2%)
Faecal calprotectin (mg/kg)	Median (IQR)	1000 (452–1900)
Age at onset		
Below 16 years	*n* (%)	15 (17.8%)
Between 16 and 40 years	*n* (%)	47 (55.9%)
Above 40 years	*n* (%)	22 (26.2%)
Disease location		
Ileum	*n* (%)	17 (20.2%)
Colon	*n* (%)	18 (21.4%)
Ileocolonic	*n* (%)	40 (47.6%)
Additional upper GI	*n* (%)	9 (10.7%)
Disease behaviour		
Inflammatory	*n* (%)	22 (26.2%)
Stricturing	*n* (%)	31 (36.9%)
Penetrating	*n* (%)	33 (39.3%)
Perianal disease	*n* (%)	23 (27.4%)
Extraintestinal manifestations	*n* (%)	42 (50%)
Prior intestinal resection	*n* (%)	46 (54.8%)
Prior perianal fistula surgical intervention	*n* (%)	23 (27.4%)
Prior treatment		
1 anti-TNF	*n* (%)	81 (96.4%)
≥2 anti-TNF	*n* (%)	58 (69.0%)
Vedolizumab	*n* (%)	42 (50.0%)
Both vedolizumab and anti-TNF	*n* (%)	39 (46.4%)
Concomitant treatment		
Oral prednisone	*n* (%)	19 (22.6%)
Thiopurine	*n* (%)	8 (9.5%)
Methotrexate	*n* (%)	2 (2.3%)

## Data Availability

The datasets used and/or analysed during the current study are available from the corresponding author upon reasonable request.

## Supplementary Materials

The following supporting information can be downloaded at: https://www.mdpi.com/article/10.3390/jcm13061539/s1, Table S1: Adverse events in patients undergoing ustekinumab therapy during the study period; Table S2: Clinical and biochemical outcomes at the fourth SC dose in patients with 12- and 8-week ustekinumab dosing intervals.
