# Supplementary material for: Association between Ustekinumab Trough Levels, Serum IL-22, and Oncostatin M Levels and Clinical and Biochemical Outcomes in Patients with Crohn’s Disease
Source: J Clin Med. 2024 Mar 7;13(6):1539. doi: 10.3390/jcm13061539 (PMC10971511; doi:10.3390/jcm13061539)
Supplement: Supplementary file 1 [file jcm-13-01539-s001.zip › jcm-2862534-supplementary.pdf]

Table S1: Adverse events in patients undergoing Ustekinumab therapy during the study period.

Adverse events (AEs) type	Patients	Number of AEs
Mild AE	10	10
Major AE	3	4
Infection	9	10
Pyelonephritis	1	2
GI infection	1	1
Perianal abscess	3	3
Abdominal abscess	3	3
Herpes Zoster	1	1
Malignancy	0	0
Headache	0	0
Dry skin/pruritis	1	1
Myalgias/arthralgia	2	2
Fatigue	0	0
Allergic reaction	1	1

Table S2: Clinical and biochemical outcomes at the fourth SC dose in patients with 12 and 8-week Ustekinumab dosing interval.

	**Q12W (*n*=21)**	**Q8W (*n* =63)**	P value
UST concentration (mean; SD; µg/mL)	1.76 ± 1.06	3.97 ± 3.36	0.376
Clinical response, n	8	20	0.160
Clinical remission, n	17	35	0.004
Steroid-free clinical remission, n	16	30	0.011
Biochemical remission, n	6	21	0.146
